# Political ideology and vaccination willingness: implications for policy design

**DOI:** 10.1007/s11077-021-09428-0

**Published:** 2021-06-16

**Authors:** Marc Debus, Jale Tosun

**Affiliations:** 1 School of Social Sciences, University of Mannheim, A5, 6, 68131 Mannheim, Germany; 2 Institute of Political Science, Heidelberg University, Bergheimer Straße 58, 69115 Heidelberg, Germany

**Keywords:** Attitudes, Beliefs, COVID-19, Europe, Ideology, Policy design

## Abstract

The COVID-19 pandemic has forced governments to impose major restrictions on individual freedom in order to stop the spread of the virus. With the successful development of a vaccine, these restrictions are likely to become obsolete—on the condition that people get vaccinated. However, parts of the population have reservations against vaccination. While this is not a recent phenomenon, it might prove a critical one in the context of current attempts to manage the COVID-19 pandemic. Consequently, the task of designing policies suitable for attaining high levels of vaccination deserves enhanced attention. In this study, we use data from the Eurobarometer survey fielded in March 2019. They show that 39% of Europeans consider vaccines to cause the diseases which they should protect against, that 50% believe vaccines have serious side effects, that 32% think that vaccines weaken the immune system, and that 10% do not believe vaccines are tested rigorously before authorization. We find that—even when controlling for important individual-level factors—ideological extremism on both ends of the spectrum explains skepticism of vaccination. We conclude that policymakers must either politicize the issue or form broad alliances among parties and societal groups in order to increase trust in and public support for the vaccines in general and for vaccines against COVID-19 in particular, since the latter were developed in a very short time period and resulted—in particular in case of the AstraZeneca vaccine—in reservations because of the effectiveness and side effects of the new vaccines.

**Supplementary Information:**

The online version contains supplementary material available at 10.1007/s11077-021-09428-0.

## Introduction

The COVID-19 pandemic has forced national governments around the world to impose major restrictions on individual freedom in order to stop the spread of the virus. These restrictions have not only sparked severe protests by groups of citizens, but also resulted in the strengthening of populist movements and their political representatives, who are generally skeptical of policies that help to stem the spread of the COVID-19 virus—a phenomenon labeled as ‘medical populism’ (Lasco, 2020). Already before the outbreak of COVID-19, vaccination was a conflictual issue, and several studies have highlighted and demonstrated empirically that the ideological orientation of citizens has a direct effect on attitudes toward vaccines.

The already existing and—possibly due to COVID-19—increasing skepticism of vaccination is likely to have a major impact on the chances of governments being able to end the restrictions on individual freedom. The implications of this skepticism are compounded by the fact that the influence of misinformation on political life and on the rationale behind implemented policies is rising (Perl et al., 2018). Only if a large majority of people are in favor of receiving one of the developed and authorized vaccines against viruses that could lead to a (global) pandemic like COVID-19 can governments retract some or even all of the restrictions imposed on individual freedom to stem the spread of the virus. It is therefore important for policymakers who are designing a vaccination strategy against viruses like COVID-19 or ones that might appear in the future to know which factors drive skepticism and opposition against vaccination.

Consequently, this contribution asks which factors have an impact on skeptical positions toward vaccines and vaccination in Europe. Because of the increasing ideological polarization in Europe over the last decade (e.g., Torgerson, 2017), which has resulted in the electoral success of left- and right-wing populist parties (e.g., Caiani & Graziano, 2019), we highlight the role of ideology in developing skeptical positions on vaccines and vaccination and argue that ideological extremism, regardless of its orientation, is likely to increase opposition toward vaccines and vaccination. On the basis of Eurobarometer survey data collected in March 2019 (European Commission, 2019), we find that—even when controlling for important individual-level factors, such as education, the economic situation, internal political efficacy, and trust in the government and the media—ideological extremism explains skepticism of vaccines and vaccination.

We take these findings as a basis for discussing implications for policy design (Capano & Howlett, 2019; Howlett & Lejano, 2013; Howlett et al., 2015; Peters et al., 2018). We suggest that political decision-makers either politicize vaccination, so that parties from the far-left or the far-right are prevented from taking ownership of the issue, or form broad alliances among parties and the societal groups they represent in order to increase trust in and public support for vaccines in general and against COVID-19 in particular. If such strategies fail and people remain skeptical of vaccines and vaccination, we argue that the risk is high that not enough people will opt to be vaccinated, meaning that a pandemic and its negative socioeconomic effects will continue for some time.

The remainder of this study unfolds as follows: The next section provides a brief review of the literature on the impact of ideology on the attitudes toward vaccines and vaccination in democracies. On that basis, we develop our expectation that ideological extremism is one of several driving forces that result in skepticism of vaccines and vaccination. “Data for evaluating the relationship between political ideology and vaccination willingness” section presents the data and the empirical strategy, while the results of the analysis—both in a descriptive and analytical manner—are shown in “Results: the more ideologically extreme, the more negative toward vaccines and vaccination” section. The final section concludes with suggestions for political decision-makers on designing a successful strategy that promotes vaccination against viruses that result in a pandemic like COVID-19.

## Ideological orientation and skepticism of vaccination

Several studies shed light on the reasons why people are skeptical of vaccines and vaccination. These studies focus on individuals’ characteristics, such as their educational, religious and psychosocial background on the one side and their trust in political institutions on the other. Several of these studies stress the impact of a person’s ideological background on their acceptance/rejection of vaccines and vaccination, but their results vary as they depend on the geographical focus of the respective study.

In a study focusing on the USA, Baumgaertner et al. (2018) demonstrate that the individuals’ ideological orientation has a strong and statistically significant effect on their attitude toward vaccines and vaccination (see also Featherstone et al., 2019). Besides trust in healthcare providers and trust in the government and medical experts, respondents who identify as conservative express less intent to be vaccinated than individuals who identify with a different ideology. This finding is robust when controlling for income and the age of the internet survey participants. A recent study that focusses on the willingness of Australians to take a COVID-19 vaccine shows that—among other factors—the support for established political parties increases the chances to take a coronavirus vaccine (Smith et al., 2021).

A further US-based survey study by Estep (2017) supports the findings by Baumgaertner et al. (2018). Estep (2017) studies the impact of the political context and one’s political ideology on vaccine acceptance in California, and finds that context matters: citizens with a conservative ideological orientation are significantly more likely to oppose vaccines, but only if they live in a county were the Democrats received high vote shares. Lin and Wang (2020) focus on the impact of personality on attitudes toward vaccination and report for the USA that, among other factors such as income and education, people with ideologically liberal orientations view vaccination as beneficial and support school vaccination. Furthermore, there is empirical evidence for the US case that parents with moderate and conservative ideological positions are less likely than liberals to report having fully vaccinated their children prior to the age of two (Rabinowitz et al., 2016).

Turning to Europe, Czarnek et al. (2020) find—on the basis of the same Eurobarometer data analyzed in this study—that the effects of right-wing ideology on vaccine attitudes are not observable in the European context. The authors contend that ideology interacts with the political interest of the participants in the survey. The more strongly people consider themselves as right-wing and the more interested they are in politics, the more likely is right-wing ideology to result in skeptical attitudes and beliefs toward vaccines and vaccination. This effect is not observable for survey participants who are less interested in politics. Furthermore, Czarnek et al. (2020) show that the substantive effects of ideology and political interest are only moderate and do not provide support for the expectation that there is a “liberal bias” against vaccines and vaccination, which suggests that, unlike in the USA, vaccines have not become a strongly politicized issue in Europe, even though national laws on mandatory vaccinations for children resulted in intense political debates, as the case of Italy in 2017 has shown (Cadeddu et al. 2020). However, the politicization of vaccination policy is likely to increase because of the conflicts between European political parties over how to handle the COVID-19 crisis. Several right-wing populist parties are campaigning against vaccination, in particular against mandatory vaccination, such as the right-wing populist “Alternative for Germany” (AfD). In France, the supporters of the far-right National Rally (NR) and the left-wing populist “Unsubmissive France” (“La France Insoumise,” LFI) reject a vaccine against COVID-19. Indeed, Ward et al. (2020) find that French who feel close to the established parties on the center-left, the center and the center-right would opt to be vaccinated against COVID-19, whereas people who identify with the far-left and the far-right parties or who do not identify with any party would reject such a vaccine (see Żuk & Żuk, 2020 for a similar result for Poland). While Cadeddu et al. (2020) find that Italians who place themselves to the right of the ideological left–right spectrum tend to consider vaccines harmful, Engin and Vezzoni (2020), by contrast, do not find an impact of political conservatism on anti-vaccination beliefs in Italy; rather, trust in political institutions and the healthcare system has a strong impact on attitudes toward vaccination.

This brief overview of the recent literature reveals that the ideological background of people seems to affect their views on vaccines and vaccination. However, while in the USA people who place themselves on the right of the ideological spectrum tend to reject vaccination or are at least skeptical of vaccines, research on European countries indicates that—if at all—ideological extremism, that is, the distance from the center of the left–right continuum, seems to affect people’s attitudes on vaccines and vaccination. On the basis of these insights, we expect Europeans of more ideologically extreme orientations to be more likely to be skeptical of vaccines.

Since the development and sale of vaccines can be highly profitable for pharmaceutical companies, some people with far-left ideological orientations have negative stances on vaccination, whereas people who locate themselves on the far right tend to reject vaccines because of their dis-identification with scientists and educational elites, their religious views, their concerns over moral purity, and their strongly hierarchal worldviews (Hornsey et al., 2020; Rabinowitz et al., 2016). Because authoritarian attitudes are a major component of the ideology of populism and its political representatives (Mudde, 2004), we should be able to observe a connection between negative positions regarding vaccines and vaccination and people who no longer feel politically represented or who do not trust key political institutions. We contend that information on individuals’ ideological stances should be taken into consideration when designing a policy that aims to attain high rates of vaccination, as is the case with the policy responses to COVID-19 or any other virus that is likely to result in a (global) pandemic.

## Data for evaluating the relationship between political ideology and vaccination willingness

The Eurobarometer data collected in March 2019 (GESIS ZA study number 7562, see European Commission, 2019) include information on the respondents’ attitudes and beliefs vis-à-vis vaccination. To evaluate whether ideological extremism both to the left and the right results in skeptical positions toward vaccines and vaccination, we rely on four indicators, which test the knowledge of people regarding vaccines and vaccination, to construct the outcome variable. More precisely, the survey participants were asked whether the following statements are true or false:

- vaccines overload and weaken the immune system;
- vaccines can cause the disease against which they protect;
- vaccines often produce serious side effects;
- vaccines are rigorously tested before being authorized for use.

If a survey respondent considered the first three statements to be true and the last one false, it indicated that he/she is skeptical of vaccines or vaccination. Since each of the statements reflects a specific aspect of vaccination, such as a vaccine’s effects or its production, we estimate four regression models with each of the variables as the respective outcome variable. Each of these outcome variables is binary, with a score of 1 indicating skeptical positions on vaccination and 0 indicating positive views of vaccination and vaccines. We estimate logistic models and control for a whole battery of factors that reflect personal characteristics of the survey respondents; these include age, gender, education, whether the respondents have children, their professional background, their trust in institutions, their satisfaction with life, whether they have experienced financial problems, whether they consider that their voice counts in the political discourse of their respective country (internal political efficacy), and their political interest.^1^

Our main explanatory variable are the individuals’ ideological beliefs. To be precise, we are interested in testing whether ideological extremism results in skeptical attitudes and positions on vaccines and vaccination; to this end, we calculate the absolute distance between the left–right self-placement of a survey respondent on the center of the left–right dimension.^2^ The greater the distance from the center, the more negative people should be toward vaccines and vaccination. To check the robustness of the findings, we estimate additional models that include the squared distance between the ideological center of the left–right dimension and the respondent’s self-placement on a left–right scale. A further model includes a measure for people who locate themselves on the far-left or the far-right of the left–right dimension. Finally, we simply include the left–right placement of the survey respondents.^3^ The latter allows us to check whether—instead of ideological extremism—a more right-wing orientation results in skeptical positions on vaccines and vaccination, as several studies on the USA suggest. The detailed results of these robustness checks are presented in online appendix, which is available here: 10.1007/s11077-021-09428-0.

## Results: the more ideologically extreme, the more negative toward vaccines and vaccination

**Fig. 1:**
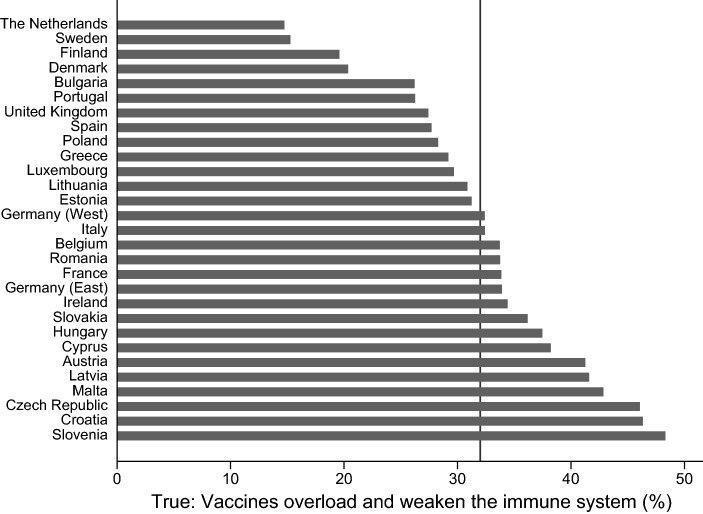
Share of respondents that agree with the statement, “vaccines overload and weaken the immune system”. Comment: Line provides information on the share of people across all countries and regions

**Fig. 2:**
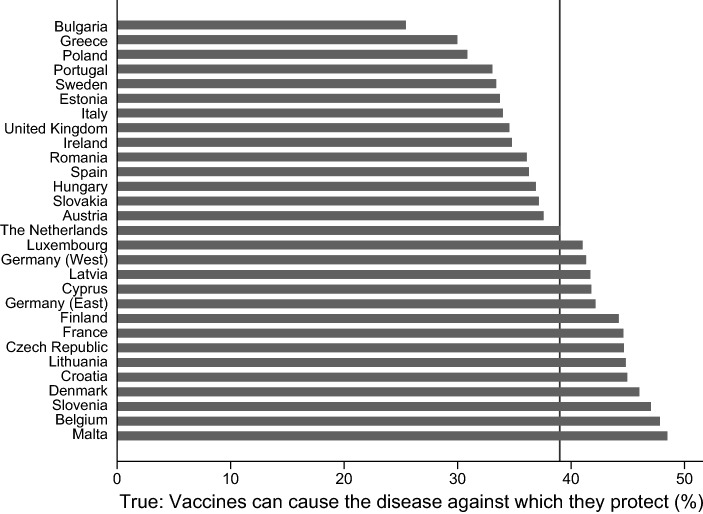
Share of respondents that agree with the statement, “vaccines can cause the disease against which they protect”. Comment: Line provides information on the share of people across all countries and regions

**Fig. 3:**
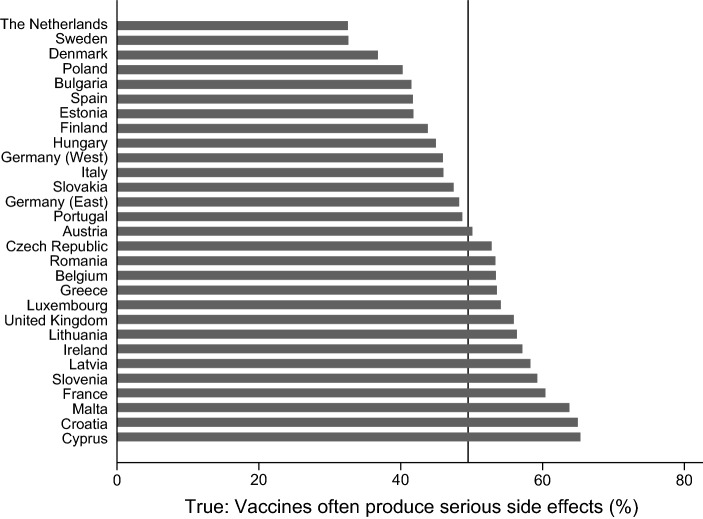
Share of respondents that agree with the statement, “vaccines often produce serious side effects”. Comment: Line provides information on the share of people across all countries and regions

**Fig. 4:**
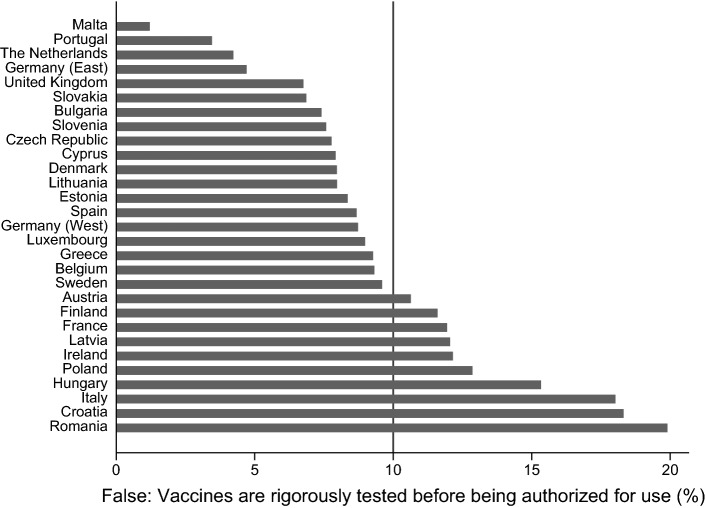
Share of respondents that disagree with the statement, “vaccines are rigorously tested before being authorized for use”. Comment: Line provides information on the share of people across all countries and regions

A significant share of people in Eastern and Western European countries have skeptical attitudes toward vaccines and vaccination. Figures 1, 2, 3, and 4 provide information on the share of respondents that agree with (incorrect) statements on vaccines or vaccination. While 32% of the respondents agree with the statement that vaccines overload and weaken the immune system, the share is significantly lower in the Netherlands and Sweden (about 15%), while in Slovenia, Croatia, the Czech Republic, and Austria more than 40% agree with the statement (see Fig. 1). Figure 2 demonstrates that almost 50% of the respondents in Malta, Belgium, and Slovenia think that vaccines can cause the disease against which they protect, while only 25% and 30% agree with this statement in Bulgaria and Greece. Across Europe, almost 40% agree with the statement that vaccines can cause those diseases which they should protect against. Roughly half of the respondents across all European countries agrees with the statement that vaccines often have serious side effects. While only one third of the Dutch and the Danes agree with that statement, almost two-thirds of the respondents from Cyprus and Croatia and 60% of the French respondents think that vaccines often have serious side effects (see Fig. 3). Only 10% of the respondents across Europe disagree with the statement that vaccines are tested rigorously before authorization. There is, again, clear variation between the countries. In Romania, Croatia, and Italy, 18–20% of the respondents think that vaccines are not rigorously tested before being authorized. The share of respondents with the same position is below 5% in the cases of Eastern Germany, the Netherlands, Portugal, and Malta (see Fig. 4).

The level of cross-country variation indicates that we need to include variables that identify the respective countries and regions when estimating the impact of ideological extremity on the respondents’ positions regarding the four indicators of vaccine-skepticism. In the following paragraphs, we present the results of the multivariate analyses, so that we can evaluate whether ideological extremism matters for skepticism of vaccines and vaccination. The results of the logistic regression models presented in Table 1 provide information on why respondents agree with the statement “vaccines overload and weaken the immune system” (model 1), “vaccines can cause the disease against which they protect” (model 2), “vaccines can cause the disease against which they protect” (model 3), and “vaccines are not rigorously tested before being authorized for use” (model 4). While we find no effect of ideological extremism for support of the statement that vaccines overload and weaken the immune system in model 1, there is evidence that people who locate themselves on the far left of the ideological spectrum are more likely to support this statement (see model 3 in Table A1). This is, however, not the case for people who consider themselves as far right-wing. There is also no evidence that with more right-wing positions people think that vaccines overload and weaken the immune system (see model 4 in Table A1).

**Table 1:** Determinants of skeptical positions on vaccination

Agreement with the following statements	“Vaccines overload and weaken the immune system”	“Vaccines can cause the disease against which they protect”	“Vaccines often produce serious side effects”	“Vaccines are not rigorously tested before being authorized for use”
*Focal explanatory variable*				
Ideological extremism	0.006	0.019^+^	0.025*	0.068**
	(0.011)	(0.011)	(0.011)	(0.017)
*Controls*				
“My voice does not count”	0.043*	0.014	− 0.007	0.220**
	(0.019)	(0.018)	(0.018)	(0.029)
Political interest strong–low	0.033^+^	0.022	0.042*	− 0.014
	(0.018)	(0.017)	(0.017)	(0.028)
Life satisfaction high–low	0.047^+^	0.047*	0.032	0.065^+^
	(0.025)	(0.024)	(0.024)	(0.038)
Tend not to trust in nat. government	0.155**	0.159**	0.199**	0.169**
	(0.036)	(0.032)	(0.033)	(0.057)
Tend not to trust in the media	0.137**	0.127**	0.216**	0.307**
	(0.034)	(0.033)	(0.031)	(0.053)
Female	0.004	0.054^+^	0.088**	− 0.036
	(0.032)	(0.030)	(0.030)	(0.049)
Years spent in the education system	− 0.040**	− 0.013^+^	− 0.039**	− 0.026*
	(0.007)	(0.007)	(0.007)	(0.011)
Respondent has children	− 0.028	− 0.022	− 0.067*	− 0.095^+^
	(0.036)	(0.034)	(0.034)	(0.055)
Age	0.001	0.014*	− 0.005	− 0.011
	(0.006)	(0.006)	(0.006)	(0.009)
Age (squared)	− 0.000	− 0.000**	0.000	0.000
	(0.000)	(0.000)	(0.000)	(0.000)
Financial problems	0.262**	0.200**	0.179**	0.260**
	(0.039)	(0.038)	(0.037)	(0.058)
Self-assessment working class	− 0.049	0.000	0.004	− 0.127^+^
	(0.042)	(0.040)	(0.039)	(0.065)
Self-assessment lower middle class	0.011	0.019	0.148**	− 0.008
	(0.045)	(0.043)	(0.043)	(0.069)
Occupation: manual worker	0.071	− 0.013	0.063	0.177**
	(0.045)	(0.043)	(0.043)	(0.068)
Unemployed	0.063	− 0.028	0.158*	− 0.033
	(0.076)	(0.073)	(0.073)	(0.117)
Retired	− 0.012	0.003	0.041	− 0.203*
	(0.055)	(0.051)	(0.051)	(0.086)
Occupation: self-employed	0.115^+^	− 0.125*	0.147*	0.175^+^
	(0.063)	(0.061)	(0.059)	(0.095)
Country dummy variables	Included	Included	Included	Included
Constant	− 0.958**	− 0.891**	0.145	− 2.824**
	(0.196)	(0.186)	(0.184)	(0.298)
*N*	20,271	20,271	20,271	20,271
*AIC*	24,295.091	26,788.444	27,205.859	12,600.181
Log likelihood	− 12,100.546	− 13,347.222	− 13,555.929	− 6253.091

Estimates from a logit model. Numbers in parentheses are standard errors. Significance levels:  ^+^*p* ≤ 0.1; *p ≤ 0.05; **p ≤ 0.01

When shifting the perspective to the statement “vaccines can cause the disease against which they protect” (see model 2 in Table 1), we find evidence that ideological extremism matters for vaccination skepticism: The more ideologically extreme respondents are, the more likely they are to think that vaccines cause diseases against which they should protect (see also model 2 in Table A2). The results of model 3 in Table A2 indicate that people from the far-right are more likely to support this statement; however, there is—again—no evidence that people are more likely to support this statement the further to the right they place themselves on a left–right dimension (model 4 in Table A2).

We find similar patterns when shifting the perspective to the statement “vaccines can often produce serious side-effects”. Here we find again that ideological extremism matters: The further away people are from the center of the left–right dimension, the more likely they are to agree with the statement that vaccines often produce serious side effects (see model 3 in Table 1 and model 2 in Table A3). Furthermore, when differentiating between people who consider themselves as far-left or far right-wing, we find that both groups of people are more likely to think vaccines often produce negative side effects (see model 3 in Table A3 in the online appendix).

Model 4 in Table 1 provides evidence that ideological extremism helps to explain why people disagree with the statement that vaccines are rigorously tested before being authorized for use. The further away people are from the center of the left–right dimension, the more likely they are to think that vaccines are not rigorously tested before authorization. Again, there is no evidence that only people who place themselves on the far-right are skeptical of testing prior to the authorization of new vaccines (see models 3 and 4 in Table A4 in online appendix).

All these results are stable, even when controlling for a battery of further variables that are usually included in empirical models when analyzing the positions and attitudes of people toward vaccines and vaccination. In line with existing studies, we find that trust in political institutions and in the media particularly matters: If people do not trust the government or the media, they are more likely to be skeptical of vaccines and vaccination. Moreover, the longer people spend in education, the less likely they are to support statements that are skeptical of vaccines and vaccination according to the findings presented here. In addition, self-reported financial problems and the feeling that one’s own views are not considered in the respective countries’ political decision-making process strengthen vaccine and vaccination skepticism. There is also evidence that a low degree of life satisfaction results in skeptical attitudes toward vaccines and vaccination, while respondents that have children seem at least less likely to agree with the statements that vaccines are rigorously tested before being authorized and that vaccines often produce serious side effects.

Overall, our findings indicate that in the case of three of the four statements regarding vaccines and the vaccination process, ideological extremism matters: the further away a respondent locates him- or herself from the center of the ideological left–right dimension, the more likely it is that he or she thinks

- that vaccines can cause the disease against which they should protect,
- that vaccines often produce serious side effects, and
- that vaccines are not rigorously tested before being authorized for use.

There is—with the exception of the statement that focused on the side effects of vaccines—no evidence that a greater right-wing orientation results in skepticism of vaccines and vaccination in the European geographical context. We therefore suggest that governments and administrations should form broad alliances and coalitions between groups and parties in society and politics in order to reduce skepticism of vaccines and the vaccination process, as this will increase the chances of more people wishing to be vaccinated against viruses that could result in a global pandemic like COVID-19. We will discuss the implications of these findings in the next section in more detail.

## Implications for designing vaccine policies

The COVID-19 pandemic has been an exceptional situation for both policymakers and the public. Several studies offer insightful discussions of how the pandemic can be studied in a meaningful manner by applying concepts and theories originating from comparative public policy (Capano et al., 2020; Weible et al., 2020) and comparative politics (Rocco et al., 2020). This literature has also elaborated on the question of how to design policy responses to COVID-19 (Capano, 2020), to which we wish to contribute by emphasizing that the ideological attitudes and beliefs of individuals are a critical factor to be considered when designing policy responses.

The question of how to design policies promoting vaccination in general and vaccines against COVID-19 in particular is particularly relevant in a phase of a pandemic when restrictions to individual freedoms are still in force and not enough people are vaccinated against a virus like COVID-19. It will not be possible to lift the restrictions imposed on individual freedom unless the herd immunity thresholds are reached. However, this could be challenging because of two main reasons. First, if a vaccine like the one against COVID-19 was developed at an unprecedented speed, people may mistrust its safety and/or effectiveness. The retracted recommendations of the AstraZeneca vaccine against COVID-19 for specific age groups are a prominent example in this regard. Second, the link established by policymakers between vaccination levels and the prospect of revoking elements of the pandemic regime may induce people to dismiss the vaccine because they feel indirectly forced to get vaccinated.

Which guidance can the above analysis and findings offer for reasonably designing vaccination policies in response to COVID-19? As the empirical analysis revealed, there are several factors that result in vaccine skepticism, including low levels of trust in political institutions, a low degree of education, and a feeling of alienation. While governments and administrations should start campaigns to inform less educated people about the huge advantages of getting vaccinated against new and well-known viruses that cause diseases, ideological extremism particularly matters in the European context for adopting skeptical views of vaccines and vaccination, in particular in times of increasing support for populist parties. In order to increase the number of people willing to be vaccinated, we consider the *processual* dimension the most relevant. This dimension concerns how individuals are informed about the vaccines and how the vaccination process and the corresponding campaign are organized. Given the changing and at times inconsistent recommendations by national agencies and the European Medicines Agency who should receive the AstraZeneca COVID-19 vaccine and in which time interval, the processual dimension seems to be a key factor for explaining the confusion and severe mistrust of the public in newly developed vaccines.﻿

One strategy for increasing vaccination willingness is to politicize the issue of vaccination itself. Ideology affects how people think of vaccines and vaccination, and some political parties may use public distrust in vaccines to maximize their support and votes in upcoming elections. Moderate parties and their representatives should bring the vaccine issue onto the political agenda, so that citizens can be persuaded of the need to be vaccinated against, for instance, COVID-19 or against other diseases like measles which emerged in the last years to some degree because of a decreasing acceptance of vaccines and vaccination. If moderate parties and their politicians depoliticize the vaccine issue, the risk is high that extremist parties will come to ‘own’ this topic, which could create a situation in which individuals supporting and trusting these parties will be beyond the reach of mainstream political forces and their (evidence-based) strategies for fighting the pandemic.

An alternative strategy for governments and administrations as well as for mainstream parties is to try to build alliances and coalitions with political parties and social groups that represent the interests of people who locate themselves on the left or the right of the ideological spectrum. This is a very challenging task, since in some European countries mainstream parties have refused to collaborate with such extremist parties on even less controversial issues than vaccination. If all political parties are capable and willing to convey the uniform message that vaccination is safe and effective, this could induce some—albeit certainly not all—individuals with extremist beliefs to overcome their reservations and to get vaccinated. This argument rests on the assumption that the credibility of an information source matters, which Jennings and Russell (2019) have shown to affect the policy addressees’ likelihood of accepting the presented information as reliable and of changing their behavior according to the respective policy. This trust stems from the individuals’ assumption that people or institutions who disseminate information share the same norms and values as they do (Smith & Mayer, 2018).

Along these lines, it further appears promising to form pro-vaccination alliances with societal actors such as religious institutions like churches, minority representatives, labor unions or welfare organizations. These actors could increase the acceptance of vaccination among individuals who are alienated from politics and do not trust any political parties and their representatives, including extremist parties. It is important to stress that there exist multiple policy options and that only some of them are top-down strategies necessary to end the pandemic as swiftly as possible. A complementary set of policy options consists of bottom-up strategies that concern the improvement of science literacy (e.g., Austin et al., 2021) and education of both youth and adults (e.g., Khubchandani et al., 2021), which pursue the goal of being prepared if a similar situation occurs in the long run.

Overall, this stage of the COVID-19 pandemic, like earlier ones, offers a valuable testing ground for examining how policymakers attempt to bring about intended changes in behavior. In this context, we contend that the politics of policy design deserves more attention when studying policy responses to COVID-19 than to more standard policy problems. The phase of vaccination in particular is predestined to conspiracy theories and misinformation, which could result in significant delays in overcoming this crisis or other pandemics which might emerge in the future. Therefore, we invite policy scientists to investigate the policy instruments adopted by governments in different countries in order to improve our understanding of how changes in behavior can be attained among individuals who display low levels of trust in democratic institutions.

## Supplementary Information

Below is the link to the electronic supplementary material.

Supplementary file1 (DOCX 39 kb)

## Footnotes

1 See the online appendix (10.1007/s11077-021-09428-0) for a description on the coding of the respective explanatory variables.

2 The left–right dimension in the Eurobarometer ranges from 1 (far left) to 10 (far right). The hypothetical center of this dimension is 5.5, meaning that ideological extremism is measured by the absolute distance between the self-placement of a survey respondent and the value 5.5.

3 Survey respondents who locate themselves with either a 1 or a 2 on the left–right axis are considered as far-left, while voters [not ‘respondents’?] who place themselves with either a 9 or a 10 are considered as far-right.
