# Supplementary material for: Political ideology and vaccination willingness: implications for policy design
Source: Policy Sci. 2021 Jun 16;54(3):477–91. doi: 10.1007/s11077-021-09428-0 (PMC8206899; doi:10.1007/s11077-021-09428-0)
Supplement: Supplementary file 1 — Supplementary file1 (DOCX 39 kb) [file 11077_2021_9428_MOESM1_ESM.docx]

**Online Appendix**

**Political ideology and vaccination willingness: implications for policy design**

**Data source**

Eurobarometer data, collected in March 2019 (GESIS ZA study number 7562). The dataset is available from the GESIS archive: https://search.gesis.org/research_data/ZA7562

The replication do-file is available upon request.

**Operationalisation of control variables**

| My voice does not count: ranges from 1 (my voice counts in my country – totally agree) to 4 (my voice counts in my country – totally disagree) |
| --- |
|  |
| Political interest strong-low: ranges from ‘strong’ (1) to ‘not at all’ (4) |
|  |
| Life satisfaction high-low: ranges from 1 (respondent very satisfied with his/her life) to 4 (respondent not at all satisfied with his/her life) |
|  |
| Tend not to trust in nat. government: 0 = tend to trust in national government; 1 = tend not to trust in media |
|  |
| Tend not to trust in the media: 0 = tend to trust in national government; 1 = tend not to trust in media |
|  |
| Female: 1 = female respondent, 0 = male respondent |
|  |
| Years spent in the education system: ranges from 1 (respondent spent up to 14 years in the education system) to 9 (respondent spent more than 22 years in the education system) |
|  |
| Respondent has children: 0 = Respondent has no children; 1 = Respondent has children |
|  |
| Age: Respondents’ age (in years) |
|  |
| Age (squared): Squared respondent’s age (in years) |
|  |
| Financial problems: 0 = Respondent has ‘almost never’ or ‘never’ financial problems; 1= Respondent has ‘from time to time’ or ‘most of the time’ financial problems |
|  |
| Self-assessment working class: Respondent does not consider him-/herself as a member of the working class (0); respondent considers him-/herself as a member of the working class (1) |
| Self-assessment lower middle class: Respondent does not consider him-/herself as a member of the lower middle class (0); respondent considers him-/herself as a member of the lower middle class (1) |
|  |
| Occupation: manual worker: 0 = Respondent is not a manual worker; 1 = respondent is a manual worker |
|  |
| Unemployed: 0 = respondent is not unemployed; 1 = respondent is unemployed |
|  |
| Retired: 0 = respondent is not retired; 1 = respondent is retired |
|  |
| Occupation: self-employed: 0 = respondent is not self-employed; 1 = respondent is self employed. |
|  |

**Descriptive statistics**

| Variable | Mean | SD | N | Min | Max |
| --- | --- | --- | --- | --- | --- |
| “vaccines overload and weaken the immune system” | .321 | .467 | 27524 | 0 | 1 |
| “vaccines can cause the disease against which they protect” | .388 | .487 | 27524 | 0 | 1 |
| “vaccines often produce serious side-effects” | .495 | .5 | 27524 | 0 | 1 |
| “vaccines are rigorously tested before being authorized for use” | .099 | .299 | 27524 | 0 | 1 |
| Ideological extremism | 1.734 | 1.372 | 22215 | .5 | 4.5 |
| Ideological extremism (squared) | 4.889 | 6.553 | 22215 | .25 | 20.25 |
| Ideological orientation: far left | .077 | .267 | 27524 | 0 | 1 |
| Ideological orientation: far right | .069 | .253 | 27524 | 0 | 1 |
| Left-right self-placement | 5.300 | 2.202 | 22215 | 1 | 10 |
| “Political interest strong-low” | 2.346 | .965 | 27426 | 1 | 4 |
| “My voice does not count” | 2.326 | .999 | 26462 | 1 | 4 |
| Life satisfaction high-low | 1.957 | .742 | 27426 | 1 | 4 |
| Tend not to trust in nat. government | .588 | .492 | 27524 | 0 | 1 |
| Tend not to trust in the media | .520 | .5 | 27524 | 0 | 1 |
| Female | .547 | .498 | 27524 | 0 | 1 |
| Years spent in the education system | 5.651 | 2.68 | 25516 | 0 | 9 |
| Respondent has children | .352 | .477 | 27524 | 0 | 1 |
| Age | 51.908 | 18.141 | 27524 | 15 | 98 |
| Age (squared) | 3023.589 | 1876.912 | 27524 | 225 | 9604 |
| Financial problems | .317 | .465 | 27524 | 0 | 1 |
| Self-assessment working class | .264 | .441 | 27524 | 0 | 1 |
| Self-assessment lower middle class | .153 | .360 | 27524 | 0 | 1 |
| Occupation: manual worker | .210 | .407 | 27524 | 0 | 1 |
| Unemployed | .052 | .221 | 27524 | 0 | 1 |
| Retired | .330 | .470 | 27524 | 0 | 1 |
| Occupation: self-employed | .069 | .254 | 27524 | 0 | 1 |

**Additional regression models**

Table A1: Determinants of agreeing with the statement, “vaccines overload and weaken the immune system”

|  | (1) | (2) | (3) | (4) |
| --- | --- | --- | --- | --- |
| *Focal explanatory variables* |  |  |  |  |
| Ideological extremism | 0.006 |  |  |  |
|  | (0.011) |  |  |  |
| Ideological extremism (squared) |  | 0.002 |  |  |
|  |  | (0.002) |  |  |
| Ideological orientation: far left |  |  | 0.114^*^ |  |
|  |  |  | (0.052) |  |
| Ideological orientation: far right |  |  | -0.029 |  |
|  |  |  | (0.056) |  |
| Left-right self-placement |  |  |  | -0.009 |
|  |  |  |  | (0.007) |
| *Controls* |  |  |  |  |
| “My voice does not count” | 0.043^*^ | 0.043^*^ | 0.044^**^ | 0.042^*^ |
|  | (0.019) | (0.019) | (0.017) | (0.019) |
| Political interest strong-low | 0.033^+^ | 0.033^+^ | 0.020 | 0.033^+^ |
|  | (0.018) | (0.018) | (0.016) | (0.018) |
| Life satisfaction high-low | 0.047^+^ | 0.047^+^ | 0.057^*^ | 0.046^+^ |
|  | (0.025) | (0.025) | (0.022) | (0.025) |
| Tend not to trust in nat. government | 0.155^**^ | 0.155^**^ | 0.142^**^ | 0.154^**^ |
|  | (0.036) | (0.036) | (0.033) | (0.036) |
| Tend not to trust in the media | 0.137^**^ | 0.137^**^ | 0.157^**^ | 0.137^**^ |
|  | (0.034) | (0.034) | (0.030) | (0.034) |
| Female | 0.004 | 0.004 | 0.021 | 0.003 |
|  | (0.032) | (0.032) | (0.029) | (0.032) |
| Years spent in the education system | -0.040^**^ | -0.040^**^ | -0.031^**^ | -0.040^**^ |
|  | (0.007) | (0.007) | (0.006) | (0.007) |
| Respondent has children | -0.028 | -0.028 | -0.019 | -0.027 |
|  | (0.036) | (0.036) | (0.032) | (0.036) |
| Age | 0.001 | 0.001 | 0.005 | 0.001 |
|  | (0.006) | (0.006) | (0.005) | (0.006) |
| Age (squared) | -0.000 | -0.000 | -0.000^*^ | -0.000 |
|  | (0.000) | (0.000) | (0.000) | (0.000) |
| Financial problems | 0.262^**^ | 0.262^**^ | 0.231^**^ | 0.262^**^ |
|  | (0.039) | (0.039) | (0.034) | (0.039) |
| Self-assessment working class | -0.049 | -0.049 | -0.012 | -0.051 |
|  | (0.042) | (0.042) | (0.037) | (0.042) |
| Self-assessment lower middle class | 0.011 | 0.011 | 0.014 | 0.009 |
|  | (0.045) | (0.045) | (0.041) | (0.045) |
| Occupation: manual worker | 0.071 | 0.071 | 0.083^*^ | 0.070 |
|  | (0.045) | (0.045) | (0.040) | (0.045) |
| Unemployed | 0.063 | 0.063 | 0.076 | 0.062 |
|  | (0.076) | (0.076) | (0.066) | (0.076) |
| Retired | -0.012 | -0.013 | 0.037 | -0.013 |
|  | (0.055) | (0.055) | (0.050) | (0.055) |
| Occupation: self-employed | 0.115^+^ | 0.115^+^ | 0.138^*^ | 0.117^+^ |
|  | (0.063) | (0.063) | (0.057) | (0.063) |
| Country dummy variables | Included | Included | Included | Included |
| Constant | -0.958^**^ | -0.958^**^ | -1.019^**^ | -0.897^**^ |
|  | (0.196) | (0.196) | (0.174) | (0.200) |
| *N* | 20271 | 20271 | 24573 | 20271 |
| *AIC* | 24295.091 | 24294.681 | 29832.377 | 24293.838 |
| Log likelihood | -12100.546 | -12100.341 | -14868.188 | -12099.919 |

Note: Estimates from a logit model. Numbers in parentheses are standard errors. Significance levels: + = p ≤ 0.1; * = p ≤ 0.05; ** = p ≤ 0.01.

Table A2: Determinants of agreeing with the statement, “vaccines can cause the disease against which they protect”

|  | (1) | (2) | (3) | (4) |
| --- | --- | --- | --- | --- |
| *Main explanatory variables* |  |  |  |  |
| Ideological extremism | 0.019^+^ |  |  |  |
|  | (0.011) |  |  |  |
| Ideological extremism (squared) |  | 0.004^+^ |  |  |
|  |  | (0.002) |  |  |
| Ideological orientation: far left |  |  | 0.035 |  |
|  |  |  | (0.050) |  |
| Ideological orientation: far right |  |  | 0.107^*^ |  |
|  |  |  | (0.053) |  |
| Left-right self-placement |  |  |  | 0.009 |
|  |  |  |  | (0.007) |
| *Controls* |  |  |  |  |
| “My voice does not count” | 0.014 | 0.013 | 0.015 | 0.013 |
|  | (0.018) | (0.018) | (0.016) | (0.018) |
| Political interest strong-low | 0.022 | 0.022 | 0.015 | 0.020 |
|  | (0.017) | (0.017) | (0.015) | (0.017) |
| Life satisfaction high-low | 0.047^*^ | 0.047^*^ | 0.059^**^ | 0.048^*^ |
|  | (0.024) | (0.024) | (0.021) | (0.024) |
| Tend not to trust in the media | 0.159^**^ | 0.159^**^ | 0.168^**^ | 0.159^**^ |
|  | (0.032) | (0.032) | (0.029) | (0.032) |
| Tend not to trust in nat. government | 0.127^**^ | 0.126^**^ | 0.132^**^ | 0.128^**^ |
|  | (0.033) | (0.033) | (0.031) | (0.033) |
| Female | 0.054^+^ | 0.053^+^ | 0.083^**^ | 0.054^+^ |
|  | (0.030) | (0.030) | (0.027) | (0.030) |
| Years spent in the education system | -0.013^+^ | -0.013^+^ | -0.008 | -0.012^+^ |
|  | (0.007) | (0.007) | (0.006) | (0.007) |
| Respondent has children | -0.022 | -0.022 | -0.034 | -0.024 |
|  | (0.034) | (0.034) | (0.031) | (0.034) |
| Age | 0.014^*^ | 0.014^*^ | 0.014^**^ | 0.015^*^ |
|  | (0.006) | (0.006) | (0.005) | (0.006) |
| Age (squared) | -0.000^**^ | -0.000^**^ | -0.000^**^ | -0.000^**^ |
|  | (0.000) | (0.000) | (0.000) | (0.000) |
| Financial problems | 0.200^**^ | 0.200^**^ | 0.176^**^ | 0.201^**^ |
|  | (0.038) | (0.038) | (0.033) | (0.038) |
| Self-assessment working class | 0.000 | -0.000 | -0.006 | 0.007 |
|  | (0.040) | (0.040) | (0.036) | (0.040) |
| Self-assessment lower middle class | 0.019 | 0.019 | -0.011 | 0.022 |
|  | (0.043) | (0.043) | (0.039) | (0.043) |
| Occupation: manual worker | -0.013 | -0.013 | -0.014 | -0.015 |
|  | (0.043) | (0.043) | (0.039) | (0.043) |
| Unemployed | -0.028 | -0.029 | -0.061 | -0.029 |
|  | (0.073) | (0.073) | (0.064) | (0.073) |
| Retired | 0.003 | 0.002 | 0.004 | 0.004 |
|  | (0.051) | (0.051) | (0.047) | (0.051) |
| Occupation: self-employed | -0.125^*^ | -0.125^*^ | -0.130^*^ | -0.128^*^ |
|  | (0.061) | (0.061) | (0.056) | (0.061) |
| Country dummy variables | Included | Included | Included | Included |
| Constant | -0.891^**^ | -0.878^**^ | -0.830^**^ | -0.908^**^ |
|  | (0.186) | (0.185) | (0.165) | (0.189) |
| *N* | 20271 | 20271 | 24573 | 20271 |
| *AIC* | 26788.444 | 26788.099 | 32475.455 | 26789.801 |
| Log likelihood | -13347.222 | -13347.050 | -16189.727 | -13347.900 |

Note: Estimates from a logit model. Numbers in parentheses are standard errors. Significance levels: + = p ≤ 0.1; * = p ≤ 0.05; ** = p ≤ 0.01.

Table A3: Determinants of agreeing with the statement, “vaccines often produce serious side-effects”

|  | (1) | (2) | (3) | (4) |
| --- | --- | --- | --- | --- |
| *Main explanatory variables* |  |  |  |  |
| Ideological extremism | 0.025^*^ |  |  |  |
|  | (0.011) |  |  |  |
| Ideological extremism (squared) |  | 0.008^**^ |  |  |
|  |  | (0.002) |  |  |
| Ideological orientation: far left |  |  | 0.103^*^ |  |
|  |  |  | (0.049) |  |
| Ideological orientation: far right |  |  | 0.186^**^ |  |
|  |  |  | (0.052) |  |
| Left-right self-placement |  |  |  | 0.012^+^ |
|  |  |  |  | (0.007) |
| *Controls* |  |  |  |  |
| “My voice does not count” | -0.007 | -0.007 | -0.003 | -0.007 |
|  | (0.018) | (0.018) | (0.016) | (0.018) |
| Political interest strong-low | 0.042^*^ | 0.042^*^ | 0.025^+^ | 0.038^*^ |
|  | (0.017) | (0.017) | (0.015) | (0.017) |
| Life satisfaction high-low | 0.032 | 0.032 | 0.032 | 0.033 |
|  | (0.024) | (0.024) | (0.021) | (0.024) |
| Tend not to trust in nat. government | 0.199^**^ | 0.198^**^ | 0.214^**^ | 0.201^**^ |
|  | (0.033) | (0.033) | (0.030) | (0.033) |
| Tend not to trust in the media | 0.216^**^ | 0.216^**^ | 0.235^**^ | 0.215^**^ |
|  | (0.031) | (0.031) | (0.028) | (0.031) |
| Female | 0.088^**^ | 0.088^**^ | 0.119^**^ | 0.088^**^ |
|  | (0.030) | (0.030) | (0.027) | (0.030) |
| Years spent in the education system | -0.039^**^ | -0.039^**^ | -0.036^**^ | -0.038^**^ |
|  | (0.007) | (0.007) | (0.006) | (0.007) |
| Respondent has children | -0.067^*^ | -0.067^*^ | -0.051^+^ | -0.071^*^ |
|  | (0.034) | (0.034) | (0.030) | (0.034) |
| Age | -0.005 | -0.005 | -0.003 | -0.005 |
|  | (0.006) | (0.006) | (0.005) | (0.006) |
| Age (squared) | 0.000 | 0.000 | 0.000 | 0.000 |
|  | (0.000) | (0.000) | (0.000) | (0.000) |
| Financial problems | 0.179^**^ | 0.179^**^ | 0.177^**^ | 0.180^**^ |
|  | (0.037) | (0.037) | (0.033) | (0.037) |
| Self-assessment working class | 0.004 | 0.002 | 0.000 | 0.012 |
|  | (0.039) | (0.039) | (0.035) | (0.040) |
| Self-assessment lower middle class | 0.148^**^ | 0.148^**^ | 0.143^**^ | 0.152^**^ |
|  | (0.043) | (0.043) | (0.039) | (0.043) |
| Occupation: manual worker | 0.063 | 0.063 | 0.083^*^ | 0.061 |
|  | (0.043) | (0.043) | (0.038) | (0.043) |
| Unemployed | 0.158^*^ | 0.157^*^ | 0.112^+^ | 0.156^*^ |
|  | (0.073) | (0.073) | (0.064) | (0.073) |
| Retired | 0.041 | 0.040 | 0.054 | 0.043 |
|  | (0.051) | (0.051) | (0.046) | (0.051) |
| Occupation: self-employed | 0.147^*^ | 0.146^*^ | 0.153^**^ | 0.143^*^ |
|  | (0.059) | (0.059) | (0.054) | (0.059) |
| Country dummy variables | Included | Included | Included | Included |
| Constant | 0.145 | 0.151 | 0.181 | 0.121 |
|  | (0.184) | (0.183) | (0.164) | (0.187) |
| *N* | 20271 | 20271 | 24573 | 20271 |
| *AIC* | 27205.859 | 27199.603 | 32976.210 | 27208.113 |
| Log likelihood | -13555.929 | -13552.802 | -16440.105 | -13557.056 |

Note: Estimates from a logit model. Numbers in parentheses are standard errors. Significance levels: + = p ≤ 0.1; * = p ≤ 0.05; ** = p ≤ 0.01.

Table A4: Determinants of disagreeing with the statement, “vaccines are rigorously tested before being authorized for use”

|  | (1) | | (2) | | (3) | | (4) | |  |  |
| --- | --- | --- | --- | --- | --- | --- | --- | --- | --- | --- |
| *Main explanatory variables* |  | |  | |  | |  | |  |  |
| Ideological extremism | 0.068^**^ | |  | |  | |  | |  |  |
|  | (0.017) | |  | |  | |  | |  |  |
| Ideological extremism (squared) |  | | 0.015^**^ | |  | |  | |  |  |
|  |  | | (0.004) | |  | |  | |  |  |
| Ideological orientation: far left |  | |  | | 0.295^**^ | |  | |  |  |
|  |  | |  | | (0.075) | |  | |  |  |
| Ideological orientation: far right |  | |  | | 0.291^**^ | |  | |  |  |
|  |  | |  | | (0.078) | |  | |  |  |
| Left-right self-placement |  | |  | |  | | -0.000 | |  |  |
|  |  | |  | |  | | (0.011) | |  |  |
| *Controls* |  | |  | |  | |  | |  |  |
| “My voice does not count” | | | 0.220^**^ | | 0.219^**^ | | 0.224^**^ | | 0.219^**^ | |
|  | | | (0.029) | | (0.029) | | (0.026) | | (0.029) | |
| Political interest strong-low | | | -0.014 | | -0.015 | | -0.020 | | -0.023 | |
|  | | | (0.028) | | (0.028) | | (0.024) | | (0.028) | |
| Life satisfaction high-low | | | 0.065^+^ | | 0.065^+^ | | 0.055^+^ | | 0.066^+^ | |
|  | | | (0.038) | | (0.038) | | (0.033) | | (0.038) | |
| Tend not to trust in nat. government | | | 0.169^**^ | | 0.169^**^ | | 0.195^**^ | | 0.168^**^ | |
|  | | | (0.057) | | (0.057) | | (0.052) | | (0.057) | |
| Tend not to trust in the media | 0.307^**^ | | 0.307^**^ | | 0.303^**^ | | 0.306^**^ | |  |  |
|  | (0.053) | | (0.053) | | (0.048) | | (0.053) | |  |  |
| Female | -0.036 | | -0.037 | | -0.014 | | -0.039 | |  |  |
|  | (0.049) | | (0.049) | | (0.045) | | (0.049) | |  |  |
| Years spent in the education system | -0.026^*^ | | -0.026^*^ | | -0.019^+^ | | -0.026^*^ | |  |  |
|  | (0.011) | | (0.011) | | (0.010) | | (0.011) | |  |  |
| Respondent has children | -0.095^+^ | | -0.095^+^ | | -0.069 | | -0.099^+^ | |  |  |
|  | (0.055) | | (0.055) | | (0.049) | | (0.055) | |  |  |
| Age | -0.011 | | -0.010 | | 0.002 | | -0.010 | |  |  |
|  | (0.009) | | (0.009) | | (0.008) | | (0.009) | |  |  |
| Age (squared) | 0.000 | | 0.000 | | -0.000 | | 0.000 | |  |  |
|  | (0.000) | | (0.000) | | (0.000) | | (0.000) | |  |  |
| Financial problems | 0.260^**^ | | 0.262^**^ | | 0.252^**^ | | 0.261^**^ | |  |  |
|  | (0.058) | | (0.058) | | (0.052) | | (0.058) | |  |  |
| Self-assessment working class | -0.127^+^ | | -0.128^*^ | | -0.111^+^ | | -0.118^+^ | |  |  |
|  | (0.065) | | (0.065) | | (0.058) | | (0.065) | |  |  |
| Self-assessment lower middle class | -0.008 | | -0.007 | | -0.035 | | -0.005 | |  |  |
|  | (0.069) | | (0.069) | | (0.063) | | (0.068) | |  |  |
| Occupation: manual worker | 0.177^**^ | | 0.176^**^ | | 0.132^*^ | | 0.173^*^ | |  |  |
|  | (0.068) | | (0.068) | | (0.061) | | (0.068) | |  |  |
| Unemployed | -0.033 | | -0.037 | | -0.070 | | -0.038 | |  |  |
|  | (0.117) | | (0.117) | | (0.102) | | (0.117) | |  |  |
| Retired | -0.203^*^ | | -0.206^*^ | | -0.166^*^ | | -0.199^*^ | |  |  |
|  | (0.086) | | (0.086) | | (0.078) | | (0.086) | |  |  |
| Occupation: self-employed | 0.175^+^ | | 0.173^+^ | | 0.165^+^ | | 0.173^+^ | |  |  |
|  | (0.095) | | (0.095) | | (0.087) | | (0.095) | |  |  |
| Country dummy variables | Included | | Included | | Included | | Included | |  |  |
| Constant | -2.824^**^ | | -2.779^**^ | | -2.988^**^ | | -2.699^**^ | |  |  |
|  | (0.298) | | (0.297) | | (0.266) | | (0.302) | |  |  |
| *N* | 20271 | | 20271 | | 24573 | | 20271 | |  |  |
| *AIC* | 12600.181 | | 12597.902 | | 15343.848 | | 12615.663 | |  |  |
| Log likelihood | -6253.091 | | -6251.951 | | -7623.924 | | -6260.832 | |  |  |

Note: Estimates from a logit model. Numbers in parentheses are standard errors. Significance levels: + = p ≤ 0.1; * = p ≤ 0.05; ** = p ≤ 0.01.
